# VURD Syndrome: Report of Three Cases

**Published:** 2013-05-14

**Authors:** Eiji Hisamatsu, Yoshikiyo Nakagawa, Yoshifumi Sugita

**Affiliations:** Department of Urology, Kobe Children’s Hospital, Kobe, Japan

**Keywords:** Posterior urethral valve, Vesicoureteral reflux, Dysplasia

## Abstract

Hoover and Duckett identified the relationship between valves, reflux, and dysplasia, commonly known as VURD (Posterior urethral valve, Unilateral vesicoureteral reflux, Renal dysplasia) syndrome. They noted preserved contralateral renal function in patients with unilateral reflux into a non-refluxing kidney. The proposed mechanism of this protection is that the refluxing collecting system acts as a pressure pop-off. Here we report three cases of VURD syndrome.

## INTRODUCTION

Hoover and Duckett introduced the concept of pressure pop-offs in the posterior urethral valves in 1982 [1]. They recognized that pressure pop-offs preserved contralateral renal function in patients with unilateral reflux into a non-functioning kidney. This phenomenon is commonly known as the valves, unilateral reflux, and renal dysplasia (VURD) syndrome. Other types of pop-off mechanism include large bladder diverticuli, urinomas, and urinary ascites [2]. In addition, Kaefer et al reported that the presence of the pop-off mechanism was a favorable prognostic sign for ultimate bladder function [3]. However, even in the presence of VURD syndrome renal failure can develop on presentation [4, 5] or after removal of the refluxing ureter and kidney, which eliminates the pop-off mechanism [6]. Here we report three cases of VURD syndrome.

## CASE REPORT

Case 1

A 7-year-old boy was referred with persistent day-time incontinence and nocturnal enuresis in addition to severe bilateral hydroureteronephrosis on ultrasonography. He had a history of sepsis at two weeks of age although the details were unknown. Prenatal ultrasonographic findings were also unknown. He required clean intermittent catheterization because of voiding difficulty and significant postvoid residual urine. Voiding cystourethrography and diuretic renography revealed that he had VURD syndrome (Fig. 1a). Subsequently, he underwent transurethral incision of the valves. On postoperative voiding cystourethrography, the unilateral reflux remained unchanged although the bladder outlet obstruction was relieved (Fig. 1b). At 9 year of age, he underwent left nephroureterectomy. After one year, clean intermittent catheterization and anticholinergic drugs were discontinued because he had neither voiding nor storing problems on video urodynamic study (Fig. 1c). The estimated glomerular filtration rate was 123 ml/m/1.73 m2 at 15 year of age. Ultrasonography revealed right hydronephrosis that was grade 2 according to the Society for Fetal Urology (SFU) classification at 17 year of age.

**Figure:**
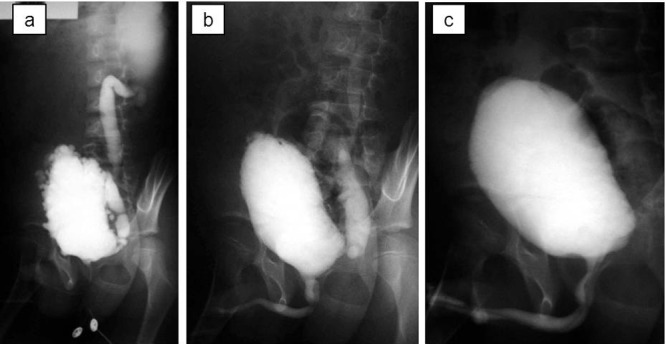
Figure 1: Voiding cystourethrography; (a) before transurethral incision of the valves, (b) before left nephroureterectomy, (c) 7 months after nephroureterectomy

Case 2

A 3-month-old boy was referred with febrile urinary tract infection (UTI) in addition to severe bilateral hydroureteronephrosis on ultrasonography. He had no history of abnormal findings on prenatal ultrasonography. Voiding cystourethrography and diuretic renography revealed that he had VURD syndrome (Fig. 2a). Subsequently, he underwent transurethral incision of the valves. The unilateral reflux remained unchanged on postoperative voiding cystourethrography (Fig. 2b). He underwent right nephroureterectomy at 3 year of age. He had been followed without anticholinergic drugs, but presented with day-time incontinence and nocturnal enuresis at 7 year of age. The bladder compliance was 10 ml/cm H2O on video urodynamic study (Fig. 2c). Day-time incontinence and nocturnal enuresis resolved after anticholinergic drugs addition. At 8 year of age, the bladder compliance increased to 30 ml/cmH2O on video urodynamic study (Fig. 2d). The estimated glomerular filtration rate was 115 ml/m/1.73 m2. Ultrasonography revealed right hydronephrosis that was SFU grade 2.

**Figure:**
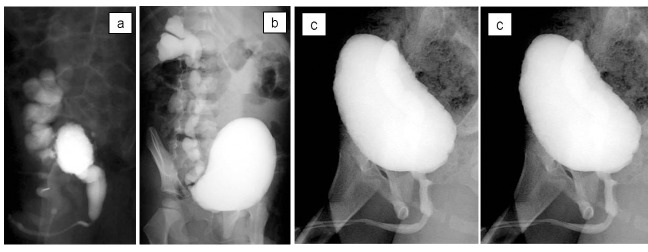
Figure 2: Voiding cystourethrography; (a) before transurethral incision of the valves, (b) before right nephroureterectomy, (c) before restart of anticholinergic drugs, (d) After restart of anticholinergic drugs

Case 3

A 7-year-old boy was referred with febrile UTI in addition to severe bilateral hydroureteronephrosis on ultrasonography. He had no history of abnormal findings on prenatal ultrasonography. Voiding cystourethrography and diuretic renography revealed VURD syndrome (Fig. 3a). Subsequently, he underwent transurethral incision of the valves. The unilateral reflux remained unchanged on postoperative voiding cystourethrography (Fig. 3b). He underwent left nephroureterectomy at 9 year of age. At 10 years of age, the bladder compliance was 17 ml/cm H2O on video urodynamic study (Fig. 3c). After that, he developed recurrent UTIs and worsening dilatation of the contralateral upper urinary tract although he continued to take anticholinergic drugs. He required clean intermittent catheterization to control UTI and prevent deterioration of contralateral kidney caused by bladder dysfunction. Right SFU grade 4 hydroureteronephrosis improved to grade 2 after the start of clean intermittent catheterization. The estimated glomerular filtration rate was 81 ml/m/1.73 m2 at 11 year of age.

**Figure:**
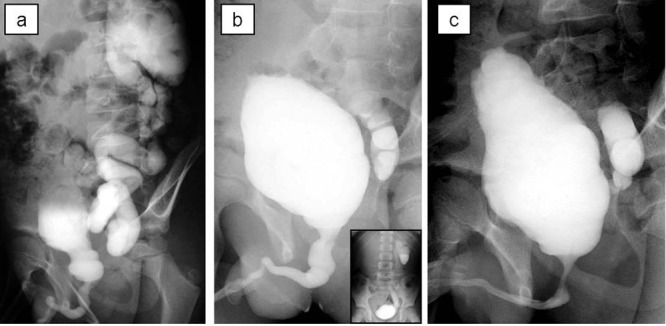
Figure 3: Voiding cystourethrography; (a) before transurethral incision of the valve, (b) before left nephroureterectomy, (c) 6 months after nephroureterectomy. Bladder trabeculation growing worse.

## DISCUSSION

According to Hoover and Duckett, nephroureterectomy should be performed in the case of a non-functioning kidney and high grade reflux to control infection and maximize normal voiding dynamics [1]. We had followed their approach for management of children with VURD syndrome although they had not developed UTI. One of our patients did not develop UTI or bladder dysfunction after removal of the refluxing ureter and kidney. On the other hand, one of the remaining two patients presented with day-time incontinence and nocturnal enuresis, which were controlled by anticholinergic drugs. Another patient developed recurrent UTIs and worsening dilatation of the contralateral upper urinary tract after removal of the refluxing ureter and kidney. He required clean intermittent catheterization to control UTI and prevent deterioration of the contralateral kidney due to bladder dysfunction.

It had been considered that patients with pop-off mechanisms have a better prognosis compared to those without it. Rittenberg et al reported that patients with one of the pop-off mechanisms were more likely to have lower nadir serum creatinine levels than those without this [2]. Kaefer et al reported that the presence of the pop-off mechanism was a favorable prognostic sign for ultimate bladder function [3]. However, the long-term protective effect of VURD has not been as helpful as originally expected. Cuckow et al concluded from long-term follow-up that renal function deteriorated in patients with VURD syndrome [7]. Narasimhan et al reported that about half of the patients with VURD syndrome had renal scarring in the contralateral kidney [8]. In addition, in Rittenberg’s report described above, one patient with VURD syndrome underwent nephroureterectomy when he was two year old. His serum creatinine progressively increased with age [2]. Similarly, in Kaefer’s report three patients with pop-off mechanisms required bladder augmentation, although the details of the pop-off mechanisms were unknown [3].

The surgical approach to a non-functioning kidney and high grade reflux has changed because the long-term prognosis is not always favorable in patients with VURD syndrome. Reinberg et al recommended that simultaneous augmentation ureterocystoplasty be considered, when nephrectomy was contemplated in a child with a history of mechanical and functional bladder obstruction [6]. Kim et al also recommended that a dilated ureter should not be removed if there was a reasonable possibility that it may be needed for bladder augmentation in the future [9]. In addition, the Philadelphia group has moved away from Duckett’s approach of an initial valve incision followed by a nephroureterectomy, except in the very unusual cases where UTI could not be controlled. They think that the pop-off mechanism places less stress on the valve bladder that has been compromised by chronically increased pressure [10].

In conclusion, one should carefully consider whether or not nephroureterectomy is necessary when managing patients with VURD syndrome. If a nephrectomy is required, leaving the severely refluxing ureter for use in subsequent reconstructive surgery is an option.

## Footnotes

**Source of Support:** Nil

**Conflict of Interest:** None declared

